# Metformin modulates immune cell infiltration into the kidney during unilateral ureteral obstruction in mice

**DOI:** 10.14814/phy2.14141

**Published:** 2019-06-27

**Authors:** Michael Christensen, Mikkel Ø. Nørgård, Michael S. Jensen, Bjarne K. Møller, Rikke Nørregaard

**Affiliations:** ^1^ Department of Clinical Medicine Aarhus University Aarhus Denmark; ^2^ Department of Clinical Immunology Aarhus University Hospital Aarhus Denmark

**Keywords:** Immune cell, metformin, STAT3, unilateral ureteral obstruction

## Abstract

Metformin is today the first choice treatment for type‐2 diabetes, but has also protective effects in several renal disease models. Previously, we have demonstrated that the protective effects in response to unilateral ureteral obstruction (UUO) are independent of organic cation transporters (OCTs), the transporters responsible for the metformin uptake into the renal cells. The mechanisms behind the renoprotective effects are incompletely understood, but our previous results indicate that the renoprotective effects at least partly could be dependent on actions of metformin outside the renal cells. In this study, we investigate whether the renoprotective effects of metformin can be mediated via systemic immunomodulatory actions. We demonstrated that metformin can affect the immune system in the kidney as well as in the peripheral blood and spleen following UUO. UUO kidneys showed infiltration of immune cells including monocytes, B cells, and T cells, but metformin limited infiltration of all cell types. UUO animals had increased spleen sizes, but this increase was attenuated by metformin. Metformin treatment surprisingly resulted in a higher proportion of monocytes with infiltratory capacity 7 days after UUO. Other studies have suggested that metformin regulates monocyte maturation through signal transducer and activator of transcription 3 (STAT3) activation, as also indicated by our results. In conclusion, our results demonstrate that metformin limits the infiltration of immune cells into the kidney, as well as modulates immune cell composition at a systemic level.

## Introduction

Since metformin was first introduced into the European market in 1958 and in the USA in 1995, clinical use as well as research of metformin have increased dramatically (Bailey 2017). Today, metformin is the preferred first‐line treatment of type‐2 diabetes, but in the last few decades numerous studies have suggested a potentially broader effect range of metformin, including reduction of cardiovascular symptoms in diabetic patients (UKPDS, 1998), reduction in risk of cancer (Evans et al. 2005) as well as treatment of polycystic ovary syndrome (Lord et al. 2003). Also, several preclinical studies have demonstrated that metformin can protect the kidney in various renal disease models (Morales et al. 2010; Alhaider et al. 2011; Takiyama et al. 2011; Neven et al. 2018). We have previously demonstrated that metformin can attenuate inflammation as well as tubular damage in a unilateral ureteral obstruction (UUO) model (Christensen et al. 2016). The same study suggested that these protective effects were independent of AMP‐activated protein kinase *β*1 (AMPK*β*1) and organic cation transporters 1 and 2 (OCT1/2), indicating that the protective effect of metformin might be systemic in the UUO model. Based on these results we hypothesize that metformin has immunomodulatory effects, and thereby protects the kidney during UUO.

Infiltration and activation of immune cells in the kidney is a hallmark in the complex pathological process following renal injury as, for example, UUO. Lin et al. (2009) elegantly demonstrated that a specific subpopulation of monocytes namely Ly6C^high^ monocytes are released from the bone marrow and recruited to the UUO kidney, where they differentiate into three subpopulations of monocytes. A similar pattern of infiltration is seen following renal ischemia–reperfusion injury (Clements et al. 2016). Monocyte ablation limits the amount of infiltration as well as fibrosis in the UUO kidney, but the interplay of monocyte subsets is complex since some subpopulations will promote inflammation and fibrosis, whereas others will promote healing of the kidney (Lin et al. 2009; Clements et al. 2016). The infiltration of immune cells are not limited to monocytes, but involves various types of leukocyte including lymphocytes and granulocytes (Lin et al. 2009). In addition, B cells have been suggested to be an important driving force of monocyte infiltration since they are one of the main sources of monocyte chemoattractant protein 1 (MCP1) in UUO kidneys (Han et al. 2017). Furthermore, B cells limit natural repair mechanisms in the postischemic kidney (Jang et al. 2010).

We and others have previously demonstrated that metformin limits inflammation in the UUO model. This has been demonstrated by, for example, lowered TNF*α* mRNA expression that of course indicate dampened inflammation, but does not explain how specific immune cells have been affected. In the current study, we tested the hypothesis that metformin affects the profile of immune cells in the kidney as well as in peripheral blood and spleen in order to test whether metformin has systemic immunomodulatory effects. This was done using multiparametric flow cytometry analysis of immune cells, in this way we were able to discriminate between individual cell types.

## Materials and Methods

### Housing and metformin treatment

All procedures were conformed in accordance with the Danish National Guidelines for care and handling of experimental animals. The protocols for animal experiments were approved by the Institute of Clinical Medicine, Aarhus University, according to the licenses for use of experimental animals issued by the Danish Ministry of Justice.

6 weeks old male mice (C57BL/6NRj) (Janvier Labs, France) had access to standard rodent diet (Altromin, Germany) and tap water. They were kept in cages with a 12‐ to 12‐h light–dark cycle in Scantainers (Scanbur, Denmark) with a temperature range of 21 ± 2°C and a humidity of 55 ± 5%. Mice were randomly divided into experimental groups. Metformin (Sigma‐Aldrich, USA) was administered in their drinking water (500/mg/kg/day). The concentration of metformin in the drinking water was estimated on an average weight of 20 g and an intake of 4.5 mL of water intake per day per mouse. The mice received metformin enriched water or normal tap water for 7 days prior to the obstruction and 3 or 7 days after the obstruction until they were sacrificed.

### Unilateral ureteral obstruction

On the day of obstruction, mice were anesthetized with sevoflurane (Abbot Scandinavia, Sweden) and injected with buprenorphine (Temgesic from Reckitt Benckiser, England). The abdomen was shaved and cleaned with ethanol wipes, eye ointment (Dechra, Denmark) was applied to protect eyes during the time of surgery, and they were placed on a heating pad to maintain normal body temperature. An abdominal incision was made to expose the left ureter and a ligature was tied around the ureter to induce the obstruction. The animals were closed in both muscle and skin before they returned to their cages where buprenorphine was administered in the drinking water for 48 h postsurgery. SHAM operated mice underwent the same procedure except the ligature around the ureter. The procedures were performed in accordance with guidelines of the animal welfare policy at Aarhus University, Denmark and approved by the Animal Experiments Inspectorate, under the Danish Veterinary and Food Administration.

### Cell preparation and flow cytometry

At the end of the experiment approx. 150 *μ*L blood was harvested from the facial vein before the mice were reopened and exsanguinated through the left ventricle of the heart. Immediately after the left kidney and spleen were removed. The kidney was decapsulated and half the kidney was frozen in liquid nitrogen and stored for later analysis. The other half was dissociated following the protocol and reagents from the Multi‐Tissue Dissociation Kit 2 (Miltenyi Biotec, Germany) and the gentleMACS Octo Dissociator with heaters (Miltenyi Biotec) before cell suspensions were passed through a 100 *μ*m cell strainer and washed in washing buffer (PBS pH 7.4 containing 0.5 % BSA and 0.09 % NaN_3_). Half the spleen was saved for later analysis and the remaining was dissociated by pressing the spleen through a 70 *μ*m cell strainer with a plunger from a 2‐mL syringe and washed. Single cell suspensions of kidney, spleen, and blood were incubated with premade antibody cocktails, all antibodies had been titrated before the experiment. (Table 1). All panels included Near IR‐Live/Dead Fix (Thermo Fisher Scientific) and leucocytes panel included Brilliant Stain Buffer (BD Biosciences, USA). For extracellular staining, samples were incubated with antibodies for 15 min before incubation with BD FACS Lysing Solution (BD Bioscences. USA). For the panel including intranuclear FOXP3 samples were permeabilized, lysed, and fixed using PerFix‐nc Kit (Beckman Coulter, USA) following the manufactures protocol. All samples were washed and resuspended in PBS before analyzed on a BD LSRFortessa (BD Biosciences) equipped with 405 nm, 488 nm, 561 nm, and 633 nm laser. BD FACSDiva^™^software was used for acquisition. Data were compensated and further analyzed in FlowJo 10 (TreeStar, USA). All data were gated according to time to exclude any abnormalities in each run and cleaned of doublets and dead cells. Samples from two animals in the 7dUUOmet group was discarded due to unsuccessful Live/Dead staining. Gating strategies are to be found at: (https://figshare.com/articles/Gating_strategies/8081123).

**Table 1:** Antibodies and fluorophores used in the analysis. Six panels were analyzed for each animals

	**Panels**	
	**Monocytes/macrophages**	**Lymphocytes**
Blood	V450‐CD11b^4^, FITC‐Ly6C^2^, APC‐CD192^2^ and PE‐CD181^4^	PerCP‐Cy5.5‐CD45^1^, FITC‐CD3*ε* 2, BV785‐CD4^1^, PE‐Cy7‐CD8^1^, PE‐eFlour610‐CD19^3^, BV605‐CD25^1^, SB436‐NK1.1^3^ and PE‐FOXp3^1^
Spleen	V450‐CD11b^4^, FITC‐Ly6C^2^, APC‐CD192^2^ and PE‐CD181^4^	PerCP‐Cy5.5‐CD45^1^, FITC‐CD3*ε* 2, BV785‐CD4^1^, PE‐Cy7‐CD8^1^, PE‐eFlour610‐CD19^3^ and SB436‐NK1.1^3^
Kidney	V450‐CD11b^4^, FITC‐Ly6C^2^ and Alexa Flour 647‐F4/80^1^	PerCP‐Cy5.5‐CD45^1^, FITC‐CD3*ε* 2, BV785‐CD4^1^, PE‐eFlour610‐CD19^3^

^1^BioLegend, USA. ^2^Miltenyi Biotec, Germany. ^3^eBioscience (Thermo Fisher Scientific). ^4^BD Biosciences, USA.

### Western blot

Renal tissue was homogenized in a dissociation buffer containing 0.3 sucrose, 25 mmol/L imidazole, 1 mmol/L EDTA, and pH 7.2 including protease inhibitors (Phosphatase Inhibitor Cocktails 2 and 3 (Sigma‐Aldrich) and Complete Mini Protease Inhibitor Cocktail Tablets (Roche). A tissuelyser LT (Qiagen, Germany) was used followed by a centrifugation. Two% SDS was added to the supernatant to prepare gel samples. Protein concentration was analyzed using a Pierce BCA protein assay kit (Roche) following manufacture's manual.

Protein samples were separated on a 12% polyacrylamide gel (Protean II; Bio‐Rad, USA) followed by a transfer to a nitrocellulose membrane (Amersham Pharmacia Biotech, UK). Nitrocellulose membranes were incubated in a 5% milk in TBS‐Tween. Membranes were washed and incubated with primary antibodies overnight at 4°C. Secondary antibodies with conjugated horseradish peroxidase were incubated for 1 h at room temperature and was visualized by chemiluminescence system (Amersham Pharmcia Biotech). Proteins of interest were normalized to total protein measured by stain‐free technology (Gürtler et al. 2013). Primary antibodies: KIM‐1 AF1817 (R&D Systems, USA), *α*‐SMA M0851 (DAKO, Denmark), HMGB1 3935S (Cell Signaling Technology, USA), IL‐33 ab54385 (Abcam, UK), STAT3, and pSTAT3 from Cell Signaling. Secondary antibodies: Goat anti‐rabbit immunoglobulin/HRP and rabbit anti‐goat immunoglobulin/HRP both from DAKO.

### Quantitative PCR

RNA was harvested from renal tissue by Nucleospin RNA II mini kit following manufacturer's protocol (Macherey Nagel, Germany). The concentration of RNA was measured by spectrophotometry at 260 nm. Synthesis of cDNA was performed using 0.5 *μ*g of RNA and the AffinityScript qPCR cDNA synthesis kit (Life Technologies, Thermo Fisher Scientific, USA). For qPCR reaction 100 ng of cDNA was used in combination with SYBR^®^ Green qPCR Master Mix according to manufacturer's protocol (Life Technologies). The reaction was performed using an Aria Mx3000p (Agilent Technologies, USA). Primers used for this project were MIP‐2 forward: CTCTCAAGGGCGGTCAAAAAGTT, MIP‐2 reverse: TCAGACAGCGAGGCACATCAGGTA.

KC Forward: GCGAATTCACCATGATCCCAGCCACCCG, KC reverse: GCTCTAGATTACTTGGGGACACCTTTTAG. 18S forward: TGTGGTGTTGAGGAAAGCAG, and 18S reverse: TCCCATCCTTCACATCCTTC.

### Statistical analysis

Statistical analysis was performed using Graph Pad Prism for Windows (GraphPad Software Inc, USA). 3dSHAM, 3dUUO, and 3dUUOmet data were analyzed using a one‐way ANOVA followed by Tukey's post hoc test for multiple comparisons between groups. 7dUUO and 7dUUOmet was analyzed using student *T*‐tests. If groups failed to meet the criteria for running a parametric test, they were analyzed by a nonparametric Mann–Whitney test.

## Results

### Metformin regulates the weight of kidney and spleen and decreases injury in the kidney

Mice received metformin in the drinking water (500 mg/kg/day) 7 days prior to UUO and until animals were sacrificed either 3 or 7 days after UUO. The body weights of the mice were not affected neither by UUO nor metformin treatment. Kidney weights were increased 3 days after UUO (3dUUO) and metformin treatment elevated the weight of the kidney both after 3dUUO and 7dUUO compared to respective untreated controls. Spleen weights increased in response to UUO, but increased less in metformin‐treated animals (Table 2).

**Table 2:** Weight measurements of mice, kidneys and spleens. 7–8 weeks old male mice, *n* = 5. Data are presented as means ± SEM

**Group**	**Body weight (g)**	**Kidney weight (g)**	**Kidney weight/body weight (×1000)**	**Spleen weight (g)**	**Spleen weight/body weight (×1000)**
3dSHAM	20.34 ± 0.57	0.12 ± 0.005	6.03 ± 0.15	0.065 ± 0.004	3.20 ± 0.13
3dUUO	19.87 ± 0.61	0.163 ± 0.005^a^	8.22 ± 0.33^a^	0.078 ± 0.0041^a^	3.94 ± 0.18^a^
3dUUOmet	18.61 ± 0.34	0.182 ± 0.005^b^	9.81 ± 0.32^b^	0.062 ± 0.002^b^	3.31 ± 0.05^b^
7dUUO	20.81 ± 0.74	0.138 ± 0.007	6.63 ± 0.12	0.094 ± 0.007	4.52 ± 0.30
7dUUOmet	19.91 ± 0.58	0.195 ± 0.006^c^	9.81 ± 0.14^c^	0.071 ± 0.005^c^	3.58 ± 0.19^c^

^a^denotes *P* < 0.05 compared with SHAM. ^b^denotes *P* < 0.05 compared with 3dUUO. ^c^denotes *P* < 0.05 compared with 7dUUO. SHAM, 3dUUO, and 3dUUOmet was analyzed by 2‐way ANOVA followed by Tukey's post hoc analysis for multiple comparisons. 7dUOO and 7dUUOmet were analyzed by Student's *t*‐test.

**Figure 1:**
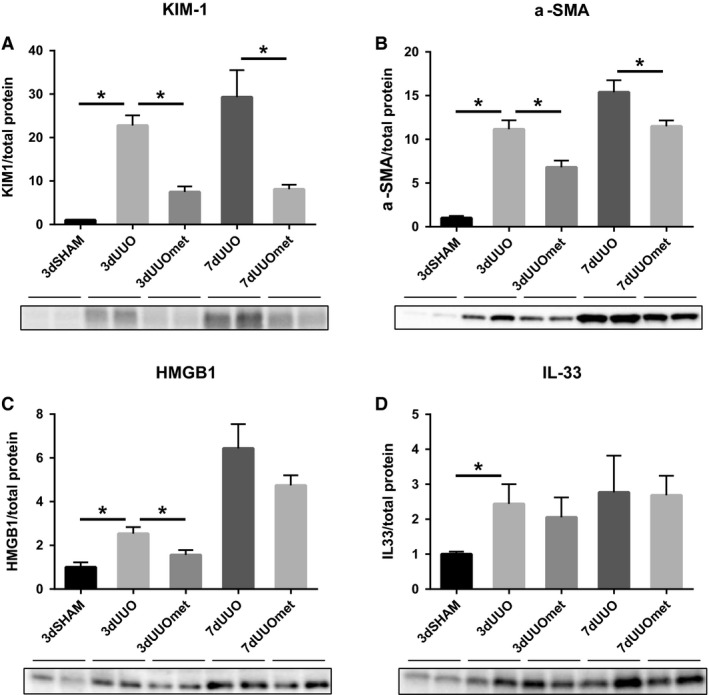
Metformin limits renal damage and profibrosis in response to UUO. (A–B) Regulation of tubular injury marker KIM‐1 and the profibrotic marker *α*‐SMA. (C–D) DAMPs regulation of HMGB1 and IL‐33. Extracts prepared from each kidney were immunoblotted with anti‐KIM‐1, *α*‐SMA, HMGB1, and IL‐33. 7–8 weeks old male mice. All extracts were run on the same gel and normalized to total protein *n* = 4/5 for each group. Each bar represents the mean ± SEM. *P* < 0.05 was considered statistically significant indicated by *. SHAM, 3dUUO, and 3dUUOmet were analyzed by 2‐way ANOVA followed by Tukey's post hoc analysis for multiple comparisons. 7dUOO and 7dUUOmet were analyzed by Student's *t*‐test.

Increased expression of kidney injury molecule‐1 (KIM‐1) and alpha‐smooth muscle actin (*α*‐SMA) was observed in UUO animals, but metformin attenuated the expression in both the 3 and 7 days UUO mice (Fig. 1A–B). Expression of damage‐associated molecular pattern molecules (DAMPs) was analyzed by high mobility group box 1 (HMGB1) and IL‐33, and both molecules showed increased expression after UUO, but only HMGB1 was reduced by metformin in 3dUUO animals (Fig. 1C–D).

### Immune cells in the kidney are decreased in numbers in metformin‐treated mice

CD11b and differential expression of Ly6C identify functionally diverse populations of monocytes/ macrophages in various tissues including the kidney (Lin et al. 2009; Clements et al. 2016; Movahedi et al. 2010).

**Figure 2:**
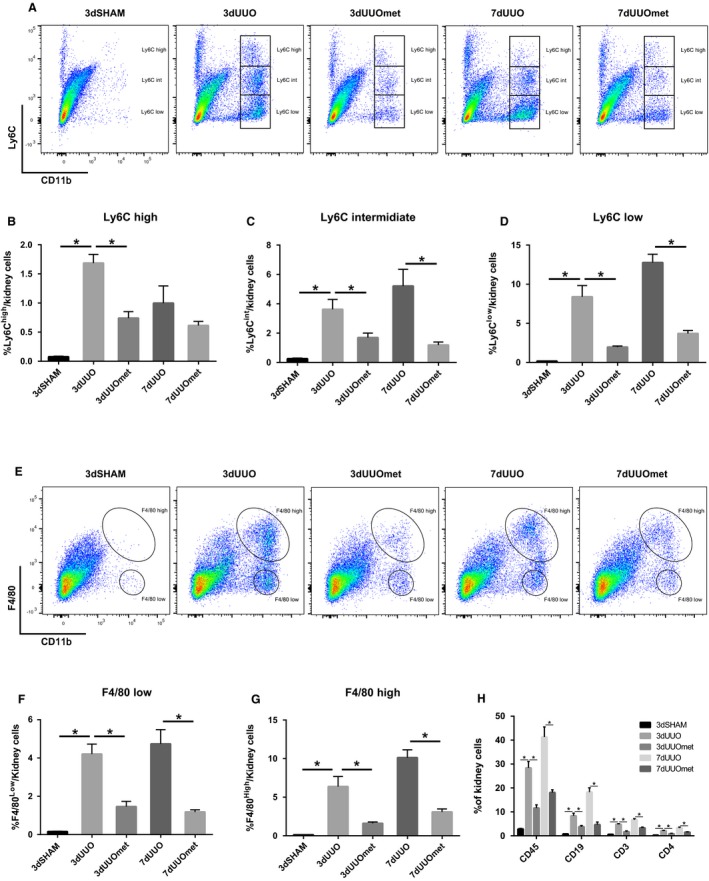
Metformin restricts immune cell infiltration into the UUO kidney. (A) Flow cytometric plots of single and live renal cells separated by the markers CD11b and Ly6C. (B–D) Graphs showing the relative proportions of the three groups of monocytes in the kidney defined by Ly6C expression. (E) FACS plots of renal cells separated by the markers CD11b and F4/80 identifying two groups of monocytes. (F–G) Graphs of the proportions of the two groups of monocytes defined by F4/80 expression. (H) Fraction of CD45, CD19, CD3 and CD4 positive cells. Seven‐ to 8‐week‐old male mice. *n*‐value = 3–5. Each bar represents the mean ± SEM. *P* < 0.05 was considered statistically significant indicated by *. SHAM, 3dUUO, and 3dUUOmet was analyzed by 2‐way ANOVA followed by Tukey's post hoc analysis for multiple comparisons. 7dUOO and 7dUUOmet were analyzed by Student's *t*‐test.

All subpopulations of CD11b^+^ cells were increased in UUO kidneys independent of their expression of Ly6C. Metformin decreased the number of Ly6C^high^ cells in 3dUUO, but not in 7dUUO. The Ly6C^int^ (intermediate) and Ly6C^low^ populations decreased in numbers after 3 and 7 days UUO in mice treated with metformin (Fig. 2A–D). CD11b and F4/80 have also been used to describe distinct populations of monocytes/macrophages by some groups and described as M1 and M2 macrophages (Clements et al. 2016). F4/80^low^ and F4/80^high^ cells were increased in 3dUUO and 7dUUO kidneys, and decreased in the corresponding metformin UUO groups (Fig. 2E–G). Furthermore, CD45^+^, CD19^+^, CD3^+^, and CD4^+^ cells were increased in renal tissue following 3dUUO and 7dUUO and decreased in response to metformin treatment (Fig. 2H).

### Monocytes and CD19^+^ cells in the blood are affected by metformin treatment

**Figure 3:**
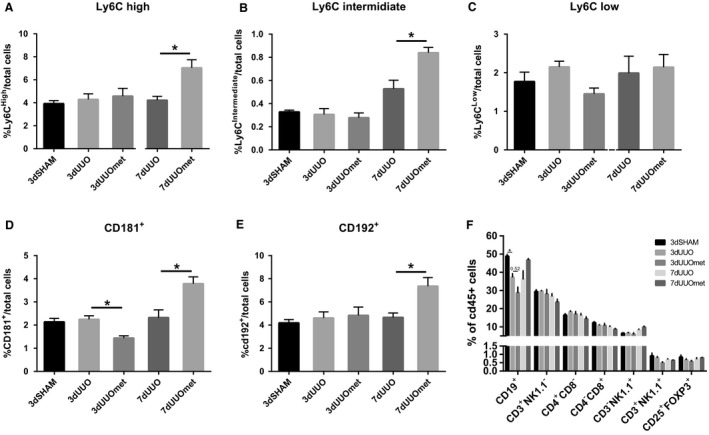
Monocyte subpopulations in the blood are affected by metformin treatment. (A–C) Graphs with the relative proportions of monocytes. (D–E) Regulation of monocytes positive for CD181 and CD192. (F) Regulation of various leucocytes in the blood. Seven‐ to 8‐week‐old male mice. *n*‐value = 3–5. Each bar represents the mean ± SEM. *P* < 0.05 was considered statistically significant indicated by *. SHAM, 3dUUO, and 3dUUOmet were analyzed by 2‐way ANOVA followed by Tukey's post hoc analysis for multiple comparisons. 7dUOO and 7dUUOmet were analyzed by Student's *t*‐test.

UUO had no effect on the monocyte profile in peripheral blood, but metformin increased the amount of Ly6C^high^ and Ly6C^int^ cells in the blood of 7dUUO mice (Fig. 3A–B). CD181^+^ cells also known as CXCR1, a receptor for an IL‐8 homolog in mice (Fan et al. 2007), was reduced in metformin‐treated 3dUUO mice compared to nontreated 3dUUO mice, but increased in 7dUUO mice subjected to metformin treatment compared to nontreated 7dUUO mice (Fig. 3D). CD192 (CCR2) is a receptor for monocyte chemoattractant protein 1 (CCL2) (Deshmane et al. 2009), and CD192^+^ cells were increased in 7dUUO metformin‐treated mice compared to 7dUUO control mice (Fig. 3E). In addition to monocytes, a selection of lymphocytes was also quantified including B cells, natural killer cells, and T cells. UUO had limited effect on these cell types in peripheral blood, and only significantly downregulated the proportion of cells positive for CD19, a pan marker for B‐cells in human and mice (Tedder and Isaacs 1989). Metformin further reduced the amount of CD19^+^ cells (*P* = 0.052) in 3dUUO (Fig. 3F).

### Metformin affects the composition of immune cells in the spleen

**Figure 4:**
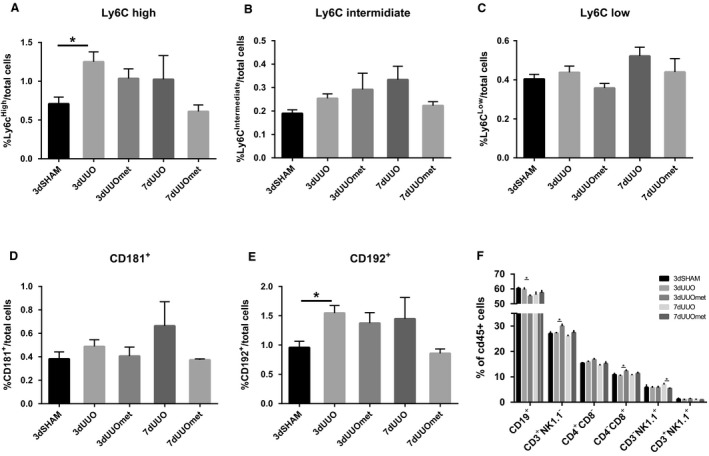
Regulation of monocytes in the spleen in response to metformin and UUO. (A–C) Graphs with the relative proportions of monocytes. (D–E) Regulation of monocytes positive for CD181 and CD192. (F) Regulation of various leucocytes in the blood. Seven‐ to 8‐week‐old male mice. *n*‐value = 3–5. Each bar represents the mean ± SEM. *P* < 0.05 was considered statistically significant indicated by *. SHAM, 3dUUO, and 3dUUOmet were analyzed by 2‐way ANOVA followed by Tukey's post hoc analysis for multiple comparisons. 7dUOO and 7dUUOmet were analyzed by Student's *t*‐test.

The spleen also works as a reservoir for immune cells to be released into the blood stream and recruited to a site of injury (Iwano and Neilson 2004; Son et al. 2014). While Ly6C^high^ and CD192^+^ cells were increased in response to 3dUUO in the spleen, there were no significant effects of metformin treatment on any monocyte subpopulations. The fractions of Ly6C^high^, Ly6C^int^, CD181^+^, and CD192^+^ cells showed numerical lower values, but the proportions of cells in 7dUUO metformin‐treated mice compared to respective UUO controls were not significantly changed (Fig. 4A–E). This is in opposition to the tendencies observed in the blood as mentioned above. The proportion of CD19^+^ cells were lower in metformin‐treated 3dUUO mice compared to nontreated 3dUUO, whereas metformin increased the proportion of CD3^+^NK1.1^−^ cells, which is explained by an increase in CD4^−^CD8^+^ cells. Furthermore, the percentage of CD3^‐^NK1.1^+^ cells was lower in 7dUUO metformin‐treated animals compared to 7dUUO controls (Fig. 4F).

### Metformin affects the STAT3 pathway and chemokine expression

**Figure 5:**
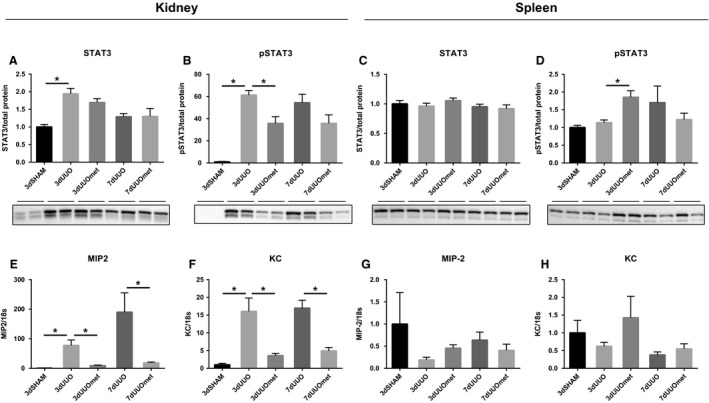
STAT3 and IL‐8 homologs are regulated by metformin treatment. (A–B) Graphs with representative blots of STAT3 and pSTAT3 in the kidney. (C–D) Graphs with representative blots of STAT3 and pSTAT3 in the spleen. Extracts prepared from each kidney and spleen were immunoblotted with anti‐STAT3 and pSTAT3. 7–8 weeks old male mice. All extracts were run on the same gel and normalized to total protein *n* = 4/5 for each group. (E–F) Graphs depicting data from qPCR with MIP2 and KC in the kidney. (G–H) Graphs showing data from qPCR with MIP2 and KC in the spleen. *n*‐value = 5. Each bar represents the mean ± SEM. *P* < 0.05 was considered statistically significant indicated by *. SHAM, 3dUUO, and 3dUUOmet were analyzed by 2‐way ANOVA followed by Tukey's post hoc analysis for multiple comparisons. 7dUOO and 7dUUOmet were analyzed by Student's *t*‐test.

In the kidney, protein expression of signal transducer and activator of transcription 3 (STAT3) and the phosphorylation form of STAT3 (pSTAT3) was increased in response to 3dUUO and administration of metformin attenuated pSTAT3 expression after 3dUUO (Fig. 5A–B). In the spleen, pSTAT3 protein expression was increased when 3dUUO mice were treated with metformin while no change was observed in STAT3 expression (Fig. 5C–D). In the kidney, the IL‐8 homologs CXCL2/MIP‐2 and CXCL1/KC were both increased at the transcriptional level in UUO animals and attenuated in metformin‐treated UUO mice. MIP‐2 and KC were not significantly regulated in the spleen neither in response to UUO nor to metformin treatment (Fig. 5E–H).

## Discussion

This study demonstrates for the first time to our knowledge that metformin limits the infiltration of immune cells into the kidney and affects the leukocyte populations at a systemic level in response to UUO. Metformin has substantial immune modulatory effects, and this study further adds knowledge to how metformin can mediate renoprotective effects in renal disease.

Increasing amounts of preclinical studies document that metformin can protect the kidney from renal injury and fibrosis, as demonstrated in diabetic kidney injury models (Takiyama et al. 2011; Alhaider et al. 2011; Ravindran et al. 2016; Zhang et al. 2017), toxin‐induced injury (Morales et al. 2010; Li et al. 2016), ischemia–reperfusion injury (Seo‐mayer et al. 2011) as well as in obstructive nephropathy (Neven et al. 2018; Christensen et al. 2016; Kim et al. 2015; Shen et al. 2016).

We have previously tested whether the effects were dependent on AMPK activation in an AMPK*β*1 KO mouse model, but found that metformin still had protective effects in these KO mice. Furthermore, metformin was also able to protect the kidneys in mice ablated for organic cation transporters 1 and 2 (OCT1/2) which can transport metformin into renal cells (Christensen et al. 2016). Together these data indicate that the renoprotective effect of metformin might be independent of AMPK and OCT1/2 in response to UUO. This leads us to the hypothesis that metformin potentially protects the kidney through systemic immune modulation.

Immune infiltration drives the deleterious progression following UUO and attenuation of infiltration will limit the formation of fibrosis (Lin et al. 2009). In this study, we found that the kidneys of metformin‐treated animals showed less signs of tubular damage demonstrated by lower expression of both KIM‐1 and the pro‐fibrotic myofibroblast marker *α*SMA, and these findings are consistent with other studies (Neven et al. 2018; Christensen et al. 2016). When analyzing specific subsets of monocytes in the renal tissue, we found increased proportions of all subpopulations of monocytes namely Ly6C^high,^, Ly6C^int^, and Ly6C^low^, in accordance with the pattern observed by others (Lin et al. 2009). Metformin significantly decreased the number of monocytes present in the kidneys and only Ly6C^high^ monocytes were unchanged in the 7dUUO metformin‐treated animals. A different approach to identify monocyte subpopulations involves the M1/M2 classification where M1 macrophages are classically activated and have an inflammatory profile, whereas M2 are alternatively activated with wound healing capacity but also a profibrotic profile (Mosser and Edwards 2008). We used the surface marker F4/80 to distinguish these two populations as done previously (Clements et al. 2016; Sogawa et al. 2017). Metformin downregulated both M1 and M2 macrophages in this study suggesting a general dampening of the innate immune response. Not only the infiltration of monocytes are affected by metformin treatment, but also T‐ and B‐lymphocytes are regulated, suggesting that the recruitment of immune cells is generally impaired.

We further analyzed the profile of immune cells of the blood as well as the spleen. We found that the spleen expanded in UUO animals, which we interpret as the result of an increase in the number of cells. Metformin‐treated UUO mice did not have larger spleens at the time of euthanization, which might indicate a dampened immune response. In this study, we did not observe any increase in the proportion of Ly6C^high^ monocytes in the blood which is in contrast to what has been described earlier in UUO mice (Lin et al. 2009). None of the populations defined by their Ly6C expression was influenced either by UUO or metformin treatment in the 3dUUO animals, only cells positive for CD181/IL‐8 receptor was decreased in 3dUUO metformin‐treated animals. This is in contrast to an increase in 7dUUO metformin‐treated animals suggesting that these cells have an infiltratory profile. This upregulation is in line with a higher proportion of Ly6C^high^, Ly6C^int^ as well as cells positive for CD192/CCR2 in metformin‐treated 7dUUO animals compared to respective UUO controls. This finding was unexpected since these subpopulations are described to have a higher ability to infiltrate damaged tissue (Lin et al. 2009) and we do not see any changes in Ly6C^high^ monocytes in the renal tissue. An explanation could be that they are accumulating in the blood because they are not recruited to the kidney due to a lower expression of, for example, chemokines MIP‐2 and KC (Fig. 5) and also MCP‐1 as we have demonstrated in an earlier study (Christensen et al. 2016). Metformin‐treated 3dUUO mice had relative fewer cells positive for CD19, a PAN marker for B cells both in the kidney, blood as well as spleen. B cells are important for the recruitment of other immune cells and the impaired recruitment of B cells into the kidney could be involved in the restriction of other immune cells (Han et al. 2017).

Metformin has in previous studies been demonstrated to have immunomodulatory effects in other disease models. This has been demonstrated in an arthritis model where metformin treatment downregulated IL‐17 producing T cells and upregulated regulatory T cells in spleens (Son et al. 2014). In the present study, we also evaluated regulatory T cells in the blood, but did not find any regulation. In an experimental autoimmune encephalomyelitis model where mice were treated with metformin, they observed a protective effect and demonstrated that metformin limited the infiltration of monocytes into the central nervous systems (Nath et al. 2009), which is in line with our observations in the kidney of the UUO animals with impaired infiltration of monocytes. In the same study, they further observed that metformin‐treated animals expressed fewer inflammatory chemokines and adhesion molecules consistent with our observations.

Signal transducer and activator of transcription 3 (STAT3) has in several cancer models been suggested to be important for the anti‐oncogene effects of metformin (Feng et al. 2014; Deng et al. 2012). Metformin has the ability to inhibit the maturation of monocytes through STAT3 inhibition (Vasamsetti et al. 2015), which also might explain the findings in our study. We therefore evaluated the STAT3 phosphorylation and found that pSTAT3 was downregulated in renal tissue, serving as a possible explanation for the reduced monocyte infiltration into the kidney.

In the present study, metformin administration in the drinking water was initiated 7 days before UUO. We have previously tested the effects of metformin both with a 7 days pre‐treatment as well as a treatment strategy where we initiated the treatment on the day of the induction of UUO. We found the most substantial effect when we used a pre‐treatment strategy, and therefore relied on that approach. However, it has previously been described that metformin had cytoprotective when the treatment was initiated 3 days before UUO (Feng et al. 2017). In that study, metformin was administrated by subcutaneous injections so we cannot directly compare our results due to differences in routes of administration.

**Figure 6:**
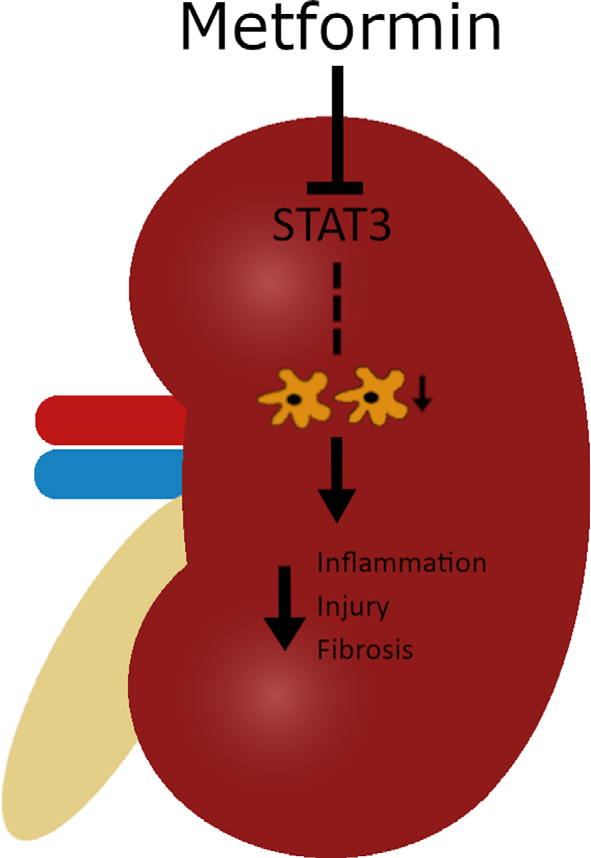
Metformin limits immune infiltration, tubular damage, and fibrosis. STAT3 is inhibited in metformin‐treated UUO kidneys, which correlates with decreased infiltration of monocytes into the UUO kidney. The decreased infiltration is associated with attenuated inflammation, injury, and fibrosis.

In conclusion, we have demonstrated that metformin treatment limits the infiltration of immune cells into the UUO kidney. Furthermore, metformin treatment has systemic effects on spleen size as well as on some leucocytes populations in the blood and spleen. STAT3 inhibition could serve as a mechanistic explanation, but further studies are needed to get a better understanding of how metformin protects the UUO kidney (Fig. 6). We believe our data describe a potentially important part of the cytoprotective effects of metformin, where metformin limits the immune filtration.

## Conflict of Interest

The authors declare no conflict of interest.
